# Circulating Angptl3 and Angptl8 Are Increased in Patients with Hypothyroidism

**DOI:** 10.1155/2019/3814687

**Published:** 2019-07-17

**Authors:** Longyan Yang, Ruili Yin, Zongwei Wang, Xiaobo Wang, Yuanyuan Zhang, Dong Zhao

**Affiliations:** Beijing Key Laboratory of Diabetes Prevention and Research, Endocrinology Center, Luhe Hospital, Capital Medical University, Beijing 101149, China

## Abstract

**Purpose:**

Angiopoietin-like proteins (Angptls) play critical roles in biological processes, primarily in lipid metabolism. The functional state of the thyroid has a profound influence on metabolism in the human body. Therefore, the aim of this study was to investigate possible changes in serum Angptl3, 4, and 8 levels in hypothyroid patients.

**Methods:**

The study included 29 patients with clinical hypothyroidism, 30 patients with subclinical hypothyroidism, and 29 healthy subjects. Baseline clinical indices, including serum thyroid function tests, were recorded and serum Angptl3, 4, and 8 levels were measured across the three groups.

**Results:**

Serum Angptl3 and 8 levels were significantly higher in the hypothyroid groups compared to the control group (*p* < 0.05). There were no differences in Angptl4 levels among the three groups (*p* > 0.05). Positive correlations were identified between Angptl3 and high-density lipoprotein cholesterol (r = 0.431,* p* < 0.001), and there was a negative correlation between Angptl3 and total tri-iodothyronine (TT3) (r = -0.220,* p* = 0.047) and free tri-iodothyronine (r = - 0.279,* p* = 0.013) levels. Angptl8 was positively correlated with triglyceride (r = 0.267,* p* = 0.012) and cholesterol levels (r= 0.235,* p* = 0.028) but was negatively correlated with tri-iodothyronine (r = -0.24,* p* = 0.031). Furthermore, we used receiver operating characteristic curve analysis to evaluate the diagnostic performance of Angptl3 and 8 in discriminating thyroid dysfunction. The area under curve for detecting thyroid dysfunction based on Angptl3 and Angptl8 was 0.763.

**Conclusions:**

Our data show that serum Angptl3 and 8 levels are increased in clinical and subclinical hypothyroid patients and that Angptl3 and 8 may serve as possible biomarkers of hypothyroid disease.

## 1. Introduction

Clinical hypothyroidism (CH) results from a lack of thyroid hormones or the inadequate actions of thyroid hormones at target tissues. Subclinical hypothyroidism (SCH) is defined as serum thyrotrophin (TSH) levels above a statistically defined upper limit of a reference range when serum free thyroxine (FT4) and free tri-iodothyronine (FT3) concentrations are at normal levels [1]. CH and SCH are the most common forms of thyroid dysfunction and are always accompanied by metabolic disorders [2] and dyslipidemia. Many studies have reported that dyslipidemia associated with CH and SCH increases the risk of endothelial dysfunction, which has been implicated in the initiation and progression of coronary artery disease [3–6]. Similarly, studies have documented that thyroid dysfunction increases the rate of cardiovascular disease [7, 8]. However, we know little about the molecular mechanisms of lipid disorders resulting from thyroid dysfunction.

Angiopoietin-like proteins (Angptls) share common protein domain characteristics with angiopoietins, which are a family of secreted glycoproteins expressed in the liver. Angptls family members include eight subtypes, Angptl1–8. Angptl3, along with Angptl4 and 8, have emerged as important regulators in lipoprotein metabolism, through the inhibition of lipoprotein lipase (LPL) [9]. Mutational studies investigating Angptl3 have shown that low plasma low-density lipoprotein cholesterol (LDL-c) decreased plasma high-density lipoprotein cholesterol (HDL-c) and low plasma triglyceride (TG) concentrations [10]. Compared with wild-type mice, Angptl4-deficient mice show lower triglyceride levels and a reduced size in atherosclerotic lesions [11]. Another study demonstrated that Angptl8-knockout rats suppressed plasma triglyceride levels and adiposity [12]. These observations suggest that Angptl3, 4, and 8 may play important roles in lipid metabolism.

Currently, some studies have indicated that adipocyte- or hepatocyte-derived metabolic regulators, such as fibroblast growth factor 19 (FGF19), fibroblast growth factor21 (FGF21), and Irisin also induce changes in patient thyroid dysfunction [13, 14].

Despite well-established associations between metabolic and thyroid function, few studies have investigated if the underlying associations between dyslipidemia and thyroid dysfunction are caused by changes in Angptls. In general, autoimmune thyroiditis has two clinical stages, which are classified according to the degree of thyroid dysfunction: subclinical hypothyroidism and hypothyroidism. In the present study, we investigate Angptl3, 4, and 8 levels in CH and SCH patients with different thyroid function statuses and interpret associations between Angptls, lipid profiles, and thyroid function indices, including thyroid hormone levels and thyroid autoantibodies.

## 2. Materials and Methods

### 2.1. Subjects

This study was performed at the Endocrine Metabolic and Immune Diseases Center at the Beijing Lu-He Hospital, affiliated to Capital Medical University, from February 2017 to November 2018. All research protocols were approved by the ethics committee from the Institutional Review Board of Capital Medical University (number 2019LH-WZ-006). All participants were informed of the study and provided written informed consent prior to participation. The study complied with the Declaration of Helsinki.

A total of 88 subjects were enrolled including age and sex-matched subjects with hypothyroidism (CH, n=29), subclinical hypothyroidism (SCH, n=30), and healthy participants (Controls, n=29). Fasted blood samples were collected from all participants. Subjects were excluded based on acute or chronic disease status, for example, diabetes mellitus, heart failure, coronary heart disease, acute-chronic renal disease, cerebrovascular event, malignancy, liver diseases, rheumatic diseases, alcohol, and smoking.

### 2.2. Diagnostic Criteria

Collected blood was allowed to coagulate at 4°C and serum was separated by centrifugation for 15 min at 3000 rpm. Serum TSH, FT4, FT3 levels, thyroid peroxidase antibody (TPOAb), thyroglobulin antibody (TgAb) levels, LDL-c, HDL-c, TC, and TG levels were measured using electrochemiluminescence immunoassay by Cobas Elecsys 601 (Roche Diagnostics, Switzerland). CH was diagnosed as serum TSH concentrations above the statistically defined upper limit of the reference range, TSH > 4.2 uIU/mL, along with a subnormal FT4 level, FT4 < 0.93 ng/dL. SCH was diagnosed as serum TSH concentrations above the statistically defined upper limit of the reference range, TSH > 4.2 uIU/mL, and serum FT4 within the normal reference range. Controls were defined by TSH, FT4, TPOAb, and TgAb within normal ranges.

### 2.3. ELISA

Serum sample aliquots were stored at -80°C until ELISA was performed. Serum angiopoietin-like proteins—Angptl3, Angptl4, and Angptl8—were measured by ELISA (Immuno-Biological Laboratories International GmBH, Japan). Experiments were conducted in compliance with manufacturer's instructions, with an intra-assay coefficient of variation (CV) of ≤ 4.8% and an interassay CV of ≤ 7.2%. All samples were performed in duplicate and repeated if the CV > 15%.

## 3. Statistical Analysis

For normally distributed variables, the data were expressed as mean ± standard deviations (SD). For nonnormally distributed variables, the data were expressed as medians with interquartile range, and categorical variables were expressed as frequencies. The Mann-Whitney rank sum test or* t*-tests were carried out according to data characteristics. Receiver operating characteristic (ROC) curve analysis was performed to assess Angptl3 and/or Angptl8 levels in predicting hypothyroid disease, and the optimal value was determined depending on the Youden index.* P* values were analyzed two-sided and when a* p* value was less than 0.05, it was considered significant. All statistical analysis were carried out using SPSS (version 22.0; SPSS Inc., Chicago, IL, USA).

## 4. Results

### 4.1. Clinical Characteristic of Study Subjects

**Figure 1:**
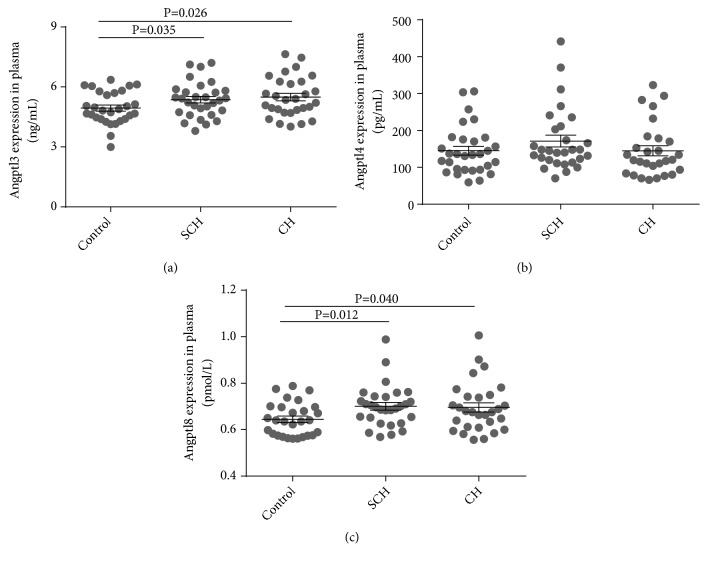
Serum Angptl3 (a), Angptl4 (b), and Angptl8 (c) levels in SCH and CH subjects. (a) Compared with healthy controls, Angptl3 levels in SCH and CH were significantly elevated; (b) Angptl4 showed no significant differences when compared with controls; (c) Serum Angptl8 was increased in the CH group when compared to the control group and was also higher in the SCH group. Data are expressed as mean ± SEM.

The characteristics of patients with CH, SCH, and controls are described (Table 1). There were no significant differences in terms of age, sex, and insulin levels among the three groups (all* p* values > 0.05). And HDL-c and TG showed no significant difference. However, LDL-c and TC levels were higher in the CH and SCH groups when compared with controls (all* p* values < 0.05). As shown in Figure 1, serum Angptl3 and 8 levels of patients with CH or SCH were significantly increased when compared with controls [Angptl3, 5.49 (5.11–5.88) vs. 4.94 (4.63–5.26), p = 0.026; 5.36 (5.03–5.69) vs. 4.94 (4.63–5.26) ng/ml, p = 0.035. Angptl8, 0.67 (0.61–0.74) vs. 0.63 (0.57–0.69), p = 0.04; 0.69 (0.64–0.74) vs. 0.63 (0.57–0.69) pmol/l, p = 0.012, respectively]. For Angptl4 levels, no significant differences were identified among the three groups [Angptl4: 144.80 (116.61–172.99) vs. 171.21 (138.54–203.89) vs. 146.89 (122.05–171.73) ng/ml].

### 4.2. Correlations among Angptl3 and 8, Thyroid Function, and Blood Lipid Indices

**Figure 2:**
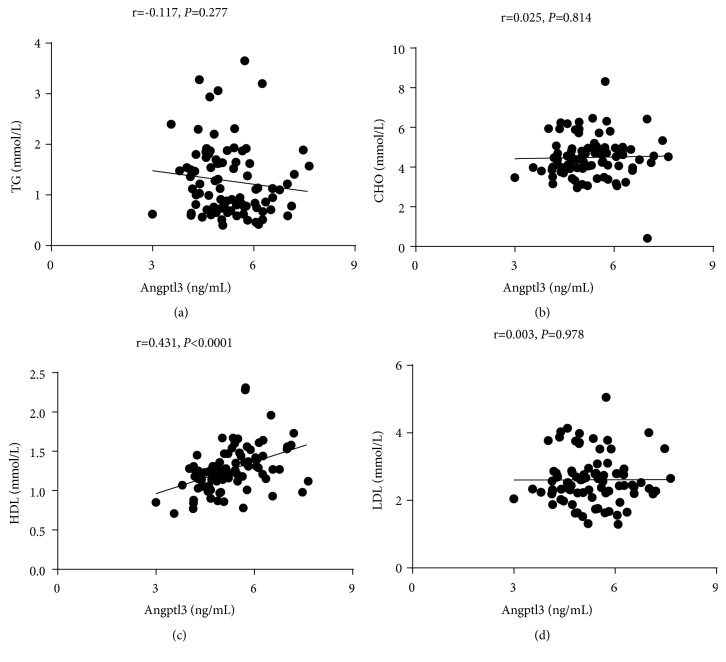
Angptl3 was positively correlated with HDL.

**Figure 3:**
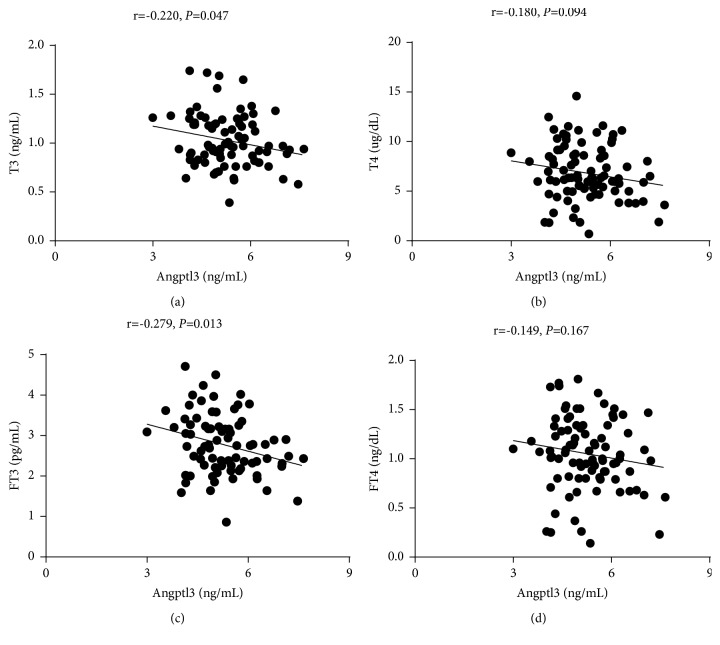
Angptl3 was negatively correlated with TT3 and FT3.

**Figure 4:**
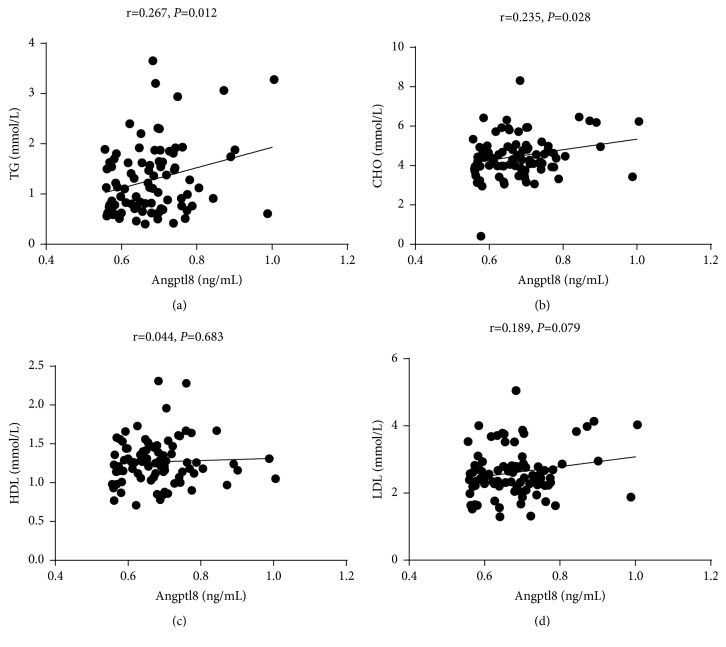
Angptl8 was positively correlated with TG and CHO.

**Figure 5:**
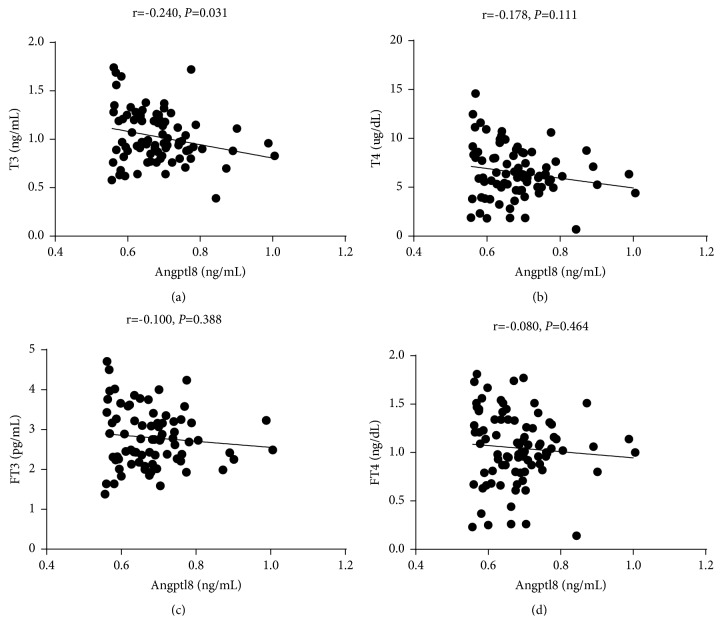
Angptl8 was negatively correlated with TT3.

To investigate the relationship between Angptl subtypes and thyroid function, we performed a correlation analysis between thyroid function, blood lipid indices, and Angptl3 and 8 plasma levels. Angptl3 was positively correlated with HDL (r = 0.431,* p* <0.001) (Figure 2), but negatively correlated with TT3 (r = -0.220,* p *= 0.047) and FT3 (r = -0.279,* p* = 0.013) (Figure 3). Angptl8 was positively correlated with TG (r = 0.267,* p* = 0.012) and CHO (r = 0.235,* p *= 0.028) (Figure 4), but negatively correlated with TT3 (r = -0.240,* p *= 0.031) (Figure 5).

**Figure 6:**
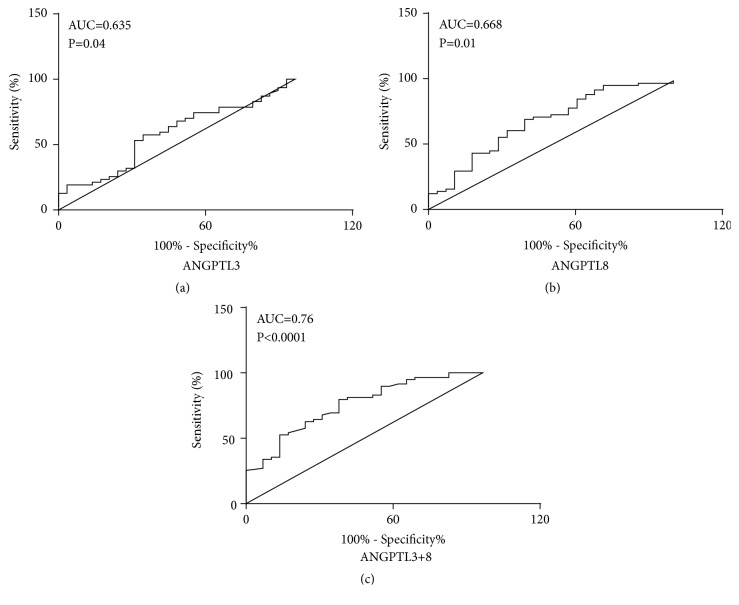
Receiver operating characteristic (ROC) analysis. (a) ROC curves for predicting SCH and CH for Angptl3. (b) ROC curves for predicting SCH and CH for Angptl8. (c) ROC curves for predicting SCH and CH for both Angptl3 and Angptl8. AUC, area under the receiver operating characteristic curve.

Both Angptl3 and Angptl8 levels appeared to be related to thyroid dysfunction, so we performed a receiver operating characteristic (ROC) curve analysis to evaluate the diagnostic performance of both Angptl subtypes in discriminating thyroid dysfunction. The area under curve (AUC) for detecting thyroid dysfunction based on Angptl3 was 0.635 (optimal cutoff value, 341.05 *μ*mol/L; sensitivity, 70.7%; specificity, 59.9%; Youden index, 0.31); and the AUC for detecting thyroid dysfunction based on Angptl8 was 0.668 (optimal cutoff value, 370.15 *μ*mol/L; sensitivity, 73.7%; specificity, 71.2%; Youden index, 0.45) (Figure 6). The AUC for detecting thyroid dysfunction based on Angptl3 and Angptl8 was 0.763 (optimal cutoff value, 370.15 *μ*mol/L; sensitivity, 73.7%; specificity, 71.2%; Youden index, 0.45).

## 5. Discussion

This study evaluated the associations between Angptl3, 4, and 8 and thyroid dysfunction. Here, we demonstrate for the first time that circulating Angptl3 and 8 levels were significantly increased in patients with CH and SCH, when compared with the control group. However, Angptl4 levels remained unchanged. Serum Angptl3 was positively correlated with HDL and negatively correlated with TT3. Due to increasing rates of metabolic disease, more and more studies have focused on their molecular elucidation. Some studies have demonstrated that multiple circulating cytokines are associated with metabolic disorders, particularly lipid and glucose diseases. Among these studies, several groups have demonstrated that Angptl3 and 8 serve as important lipid metabolism factors closely associated with metabolic disease. A study by Abu-Farha* et al.* suggested that Angptl3, 4, and 8 levels are increased in obese and T2DM patients [15]. Simultaneously, a Japanese study showed that circulating Angptl4 was significantly higher in patients with impaired glucose metabolism and that a deficiency in Angptl4 could improve lipid metabolism and protect against atherosclerosis [16]. Güneş M* et al.* suggested there was a significant increase in Angptl4 levels in polycystic ovary syndrome (PCOS) when compared to healthy subjects and that changes in PCOS may be related to insulin resistance [17]. It has also been suggested that Angptl8 is positively correlated with hepatocellular lipid content, independent of obesity and insulin resistance [18]. Angptl8 was positively correlated with circulating triglycerides and LDL-c levels and was inversely correlated with circulating HDL levels in populations undergoing routine medical checkups [16].

The hepatic overexpression of Angptl8 causes hypertriglyceridemia and increased insulin secretion. In addition, Angptl8-knockout mice have reduced adipose tissue and lower triglyceride levels when compared to wild-type mice [19]. At the same time, in high-risk cohorts with morbid obesity or type 2 diabetes, circulating Angptl8 levels correlate significantly with atherogenic lipid profiles [20]. Furthermore, Haller and colleagues further clarified that Angptl8 inhibited LPL activity and induced plasma triglyceride levels in the presence of Angptl3 [21]. Together, these results indicate that the Angptl family may be promising regulators in glucose and lipid metabolism and may play important roles in metabolic disease.

CH and SCH are associated with some metabolic disorders including insulin resistance or high insulin levels, dyslipidemia, obesity, and endothelial dysfunction [22–24], which are closely associated with atherosclerosis and cardiovascular risk. A meta-analysis of 55,287 participants indicated that SCH is associated with an increased risk of coronary heart disease (CHD) and mortality in those with higher TSH levels, in particular in those with TSH levels at 10 mIU/L or greater [25]. Some studies have shown that some adipokines or hepatokines, such as Irisin and FGF21 levels, are altered in thyroid dysfunction [14, 26, 27]. Because the associations between Angptl and SCH or CH are not clear, we sought to understand the potential role of Angptl3, 4, and 8 on these diseases. In our study, Angptl3 and 8 levels were increased when compared to the control group. This is consistent with a previous study, in which Angptl 8 levels were also increased in SCH and CH groups [28]. Furthermore, another study reported that thyroid hormones can suppress Angptl3 mRNA in a TR*β*-dependent manner, but not Angptl4 mRNA [29]. Similarly, a negative correlation between Angptl3 and TT3 and FT3 was observed in our study. It is therefore possible that lower thyroid hormones in CH patients are accompanied by high Angptl3 levels.* In vivo* and* in vitro* studies have indicated that Angptl3 must form a complex with Angptl8 to inhibit LPL [30, 31]. Angptl8 promotes the cleavage of Angptl3 and therefore increases Angptl3 activity [32]. In our study, Angptl8 was positively correlated with TG and CHO. This observation may partly explain the high dyslipidemia levels reported in this study: elevated circulating Angptl3 and 8 levels in CH and SCH patients resulted in greater inhibition of LPL. We also observed that Angptl4 levels were not altered in the CH or SCH patients when compared to the controls. On the one hand, this may have been because thyroid hormones successfully suppressed Angptl3 mRNA, but not Angptl4 mRNA. However, this effect may have been mechanistic; Angptl3 and Angptl4 affect LPL via separate mechanisms, whereby Angptl4 promotes LPL inactivation whereas Angptl3 suppresses lipase catalytic activity [33].

Furthermore, by using ROC analysis, we confirmed that Angptl3 and Angptl8 could predict thyroid dysfunction. Overall, we have shown that Angptl3 and 8 could be promising biomarkers and therapeutic targets for thyroid dysfunction.

There are limitations to our study. Firstly, it was cross-sectional, and it only addressed associations between changes in circulating Angptl3 and 8 and thyroid dysfunction. Changes in serum Angptl 3 and 8 levels after chronic replacement of thyroid hormones were not assessed. Furthermore, other factors such as adipokines or hepatokines, hormones, or other parameters that may have affected Angptl 3 and 8 levels were not examined. In addition, the sample size was small. Based on these limitations, more large-scale population studies are needed to understand the relationship between Angptl and thyroid dysfunction.

In summary, our study demonstrated that circulating Angptl3 and Angptl8 levels are increased in patients with CH and SCH. Thus, it appears that thyroid insufficiency may impact on circulating Angptl3 and 8 levels. To highlight the significance of Angptl subtypes in metabolic disease pathogenesis, further studies are required. These studies could elucidate the role of Angptl subtypes in hypothyroidism and uncover mechanistic connections between thyroid dysfunction and metabolic disease.

## Figures and Tables

**Table 1:** Study population information.

Group	Healthy control	Subclinical hypothyroidism	Overt hypothyroidism	*p* value
Sex (F/M)	29 (16/13)	30 (23/7)	29 (24/5)	0.05
Age (year)	43±11	44±13	44±15	0.43
T3 (ng/mL)	1.30 (1.20-1.41)	0.95 (0.87-1.02)	0.89 (0.82-0.97)	<0.001
T4 (ug/dL)	9.78 (1.8-2.76)	6.61 (6.26-6.97)	4.13 (3.49-4.77)	<0.001
FT3 (pg/mL)	3.60 (3.33-3.86)	2.82 (2.64-3.00)	2.16 (1.98-2.35)	<0.001
FT4 (ng/dL)	1.39 (1.30-1.48)	1.07 (1.01-1.13)	0.70 (0.58-0.81)	<0.001
TSH (uIU/mL)	2.46 (1.8-2.76)	14.19 (10.36-19.07)	16.48 (9.86-45.17)	<0.01
TPOAb (U/mL)	18.71(15-22.79)	177.45 (23.26-236.68)	117.45 (57.69-352.23)	<0.01
TgAb (U/mL)	16.08 (12.18-30.69)	468.6 (206.2-557.5)	437.15 (234.13-1326.2)	<0.01
Insulin (pmol/L)	10.44±4.82	10.69±7.54	11.58±8.77	>0.05
HDL-C (mmol/L)	1.15 (1.07-1.22)	1.41 (1.28-1.53)	1.25 (1.15-1.35)	>0.05
LDL-C (mmol/L)	2.33(1.77-2.59)	2.58(2.21-2.91)	2.73 (2.44-3.62)	<0.001
TG (mmol/L)	1.01 (0.83-1.19)	1.45 (1.16-1.73)	1.37 (1.08-1.66)	>0.05
TC (mmol/L)	4.00 (3.76-4.24)	4.58 (4.10-5.06)	4.87 (4.51-5.24)	<0.05
Angptl3 (ng/mL)	4.94 (4.63-5.26)	5.36 (5.03-5.69)	5.49 (5.11-5.88)	<0.05
Angptl4 (pg/mL)	144.80 (116.61–172.99)	171.21 (138.54–203.89)	146.89 (122.05–171.73)	>0.05
Angptl8 (pmol/L)	0.63(0.57-0.69)	0.69 (0.64-0.74)	0.67(0.61-0.74)	<0.05

F: female; M, male.

a. Data are expressed as mean±SD.

b. Data are expressed as median with interquartile range.

## Data Availability

All data generated or analyzed during this study are included in this published article.
